# Screensaver: an open source lab information management system (LIMS) for high throughput screening facilities

**DOI:** 10.1186/1471-2105-11-260

**Published:** 2010-05-18

**Authors:** Andrew N Tolopko, John P Sullivan, Sean D Erickson, David Wrobel, Su L Chiang, Katrina Rudnicki, Stewart Rudnicki, Jennifer Nale, Laura M Selfors, Dara Greenhouse, Jeremy L Muhlich, Caroline E Shamu

**Affiliations:** 1 ICCB-Longwood/NSRB Screening Facility, Harvard Medical School, 250 Longwood Avenue, Boston, MA 02115, USA; 2 Broad Institute, 7 Cambridge Center, Cambridge, MA 02142, USA; 3 Department of Systems Biology, Harvard Medical School, 200 Longwood Ave. Boston MA 02115, USA

## Abstract

**Background:**

Shared-usage high throughput screening (HTS) facilities are becoming more common in academe as large-scale small molecule and genome-scale RNAi screening strategies are adopted for basic research purposes. These shared facilities require a unique informatics infrastructure that must not only provide access to and analysis of screening data, but must also manage the administrative and technical challenges associated with conducting numerous, interleaved screening efforts run by multiple independent research groups.

**Results:**

We have developed Screensaver, a free, open source, web-based lab information management system (LIMS), to address the informatics needs of our small molecule and RNAi screening facility. Screensaver supports the storage and comparison of screening data sets, as well as the management of information about screens, screeners, libraries, and laboratory work requests. To our knowledge, Screensaver is one of the first applications to support the storage and analysis of data from both genome-scale RNAi screening projects and small molecule screening projects.

**Conclusions:**

The informatics and administrative needs of an HTS facility may be best managed by a single, integrated, web-accessible application such as Screensaver. Screensaver has proven useful in meeting the requirements of the ICCB-Longwood/NSRB Screening Facility at Harvard Medical School, and has provided similar benefits to other HTS facilities.

## Background

High throughput screening (HTS) technologies are commonly understood to encompass assays that generate tens of thousands of data points, usually acquired in 96-, 384-, or 1536-well microplate format. Use of HTS technologies is becoming standard in academe to identify small molecule research tools and to support genome-scale RNAi screening [1-3]. High throughput screening facilities are expensive to establish and maintain due to the cost of laboratory automation, screening libraries, and personnel. Thus, many organizations create economies of scale by providing shared "core" HTS facilities to support multiple screening projects run by multiple research groups. Such facilities require an informatics infrastructure that supports access to and analysis and sharing of experimental data.

In the case of the ICCB-Longwood/NSRB screening facility [4] and [5] at Harvard Medical School, which was one of the first high throughput screening facilities established in academe, the informatics infrastructure had developed into an ad hoc, divergent set of independent web applications, databases, and file repositories. To address the diverse experiment- and administrative-based requirements of the facility, while simultaneously addressing the need to unify its data storage and access infrastructure, the ICCB-Longwood/NSRB facility initiated development of the Screensaver web application.

**Figure 1:**
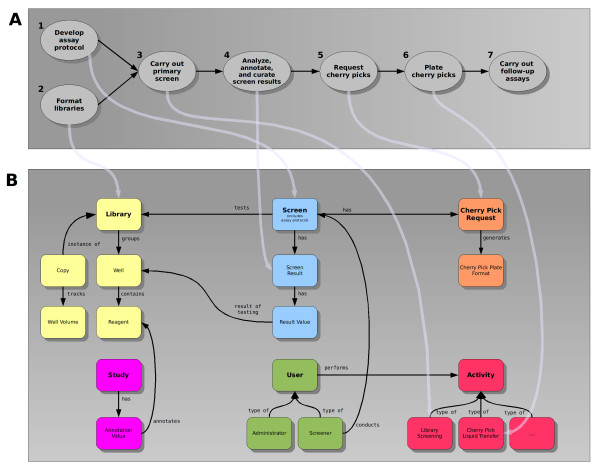
**Screening Workflow and Screensaver Domain Model**. A - Screening Workflow (mapped onto Screensaver Domain Model): A typical screening workflow is represented at the top of the figure (A). The light gray lines indicate how data generated from the workflow map onto the Screensaver domain model (B). The screener develops an assay protocol (1) that is entered into Screensaver by administrators and stored as a Screen entity. The screening facility provides the library stock plates (2) to carry out the primary screen (3). Library information, imported into Screensaver by administrators, is stored as Library entities. The screener and facility staff carry out data analysis, annotation, and curation (4) before the data is deposited into the database by administrators. Based upon the analysis, the screener chooses library reagents for follow-up work and requests the cherry picks (5), which are entered into Screensaver by administrators. Administrators then use Screensaver to generate plate mapping files for cherry pick plate creation. The mapping files are employed by facility staff who use laboratory automation equipment to plate the cherry picks (6). Cherry pick plates are screened in secondary and tertiary assays by the screener (7). B - Screensaver Domain Model (primary entities): The primary entities of the Screensaver domain model are represented at the bottom of the figure (B) and are partitioned into color-coded groups. Two primary types of users are supported: Administrators and Screeners. Administrators represent the staff working at the facility. Screeners are users that conduct Screens. Screens, in turn, test the Reagents in one or more Libraries using protocols specified in the Screen. The Screen produces Screen Results containing a set of Result Values. Each Result Value is a single experimental data point for a given Library Well. A Study contains Annotation Values, which annotate Reagents with biologically relevant information, derived from a Screen or imported from other sources, such as journal publications or public databases. A Library groups together a set of Wells and Reagents, laid out across one or more plates. A Copy represents a physical instance of a Library and its plates, and tracks the volumes of reagent remaining in each of its plates' wells. Activities track the events occurring in the lab as well as data updates made in the database (for auditing purposes), and are associated with the User that performed the activity. Cherry Pick Requests are created for a given Screen when cherry pick plates need to be produced for a follow-up validation assay.

Screensaver aids an HTS facility by managing the data for its libraries and reagents, its screening experiments, along with the screeners and daily activities associated with these experiments, the generated raw and annotated data, and the follow-up requests for reagents to be cherry picked from library stock plates. A sample screening workflow and its interface with Screensaver is diagrammed in Figure 1A. Additionally, Screensaver allows users to perform cross-screen comparisons across screen result data and third-party annotation data, which is available from databases such as PubChem [6], DRSC [7], GenomeRNAi [8], ChEMBL [9], and ChemBank [10].

## Implementation

Screensaver is a database web application written in the Java programming language. The software has been developed using free, open source software technologies, including the PostgreSQL relational database system [11], Hibernate [12], Spring [13], Apache MyFaces [14], Facelets [15], and the Apache Tomcat servlet engine [16]. The system's architecture thus incorporates technologies and design techniques that are intended to facilitate rapid modification of the software, including a clearly-defined domain model implementation, an object-relational mapping framework, a component-based web user interface, and a dependency injection framework. These architectural aspects, described below in more detail, have helped the developers update the software in response to the quickly evolving informatics requirements of our HTS facility. The architecture has also benefited other HTS facilities that have already adapted Screensaver for their own use (as detailed in "Current Usage" section).

### Domain Model

Screensaver employs a domain-driven design [17] where the conceptual domain model [18] upon which it based is explicitly represented within the structure of the software. The domain model layer forms the foundation of the Screensaver application and is comprised of entity definitions (Screen, Library, etc.) that specify the properties, relationships, constraints, and operations for each entity type. Meta data, in the form of Java annotations, are also specified on each entity and used by Hibernate to map the entity properties and relationships to a physical database schema, which can then be automatically generated. As Hibernate provides the implementation of all persistence operations, changes made to the domain model do not require developers to update database access code. Most service-layer and presentation-layer dependencies upon the domain model can be detected at compile-time, so that the impact of domain model changes can be reliably determined and addressed by developers.

Since the domain model is defined as a set of Java-based entity classes whose persistence behavior is automatically managed, custom data import and export utilities can be readily developed without writing database-specific code or SQL statements. Import and export routines can be written directly in terms of the Java entity classes that represent the data needed. At ICCB-Longwood/NSRB, we found this design to be a significant help during the task of migrating data from the legacy systems that were in use at our facility. Other facilities that need to migrate data into or out of Screensaver should realize similar benefits.

High-level, facility-specific operations that manipulate the domain model entities are placed into separate service, policy, and input/output layers. This design allows the domain model layer to remain a canonical representation that can be more readily adapted for use in new Screensaver deployments.

The primary entities of the Screensaver domain model are highlighted in Figure 1B and are described in more detail in the Results and Discussion section, below.

### Web Application Technologies

The Screensaver web application is deployable to a Tomcat 5.5 servlet engine. Use of the Spring application framework provides Screensaver with the necessary "enterprise services," including database connectivity and transaction management. The Spring Framework technology also provides Screensaver with a high-level, XML-based application configuration, offering a mechanism by which Screensaver can be customized for new deployments. For example, an alternate data access policy or user authentication mechanism can be specified with minimal effort. The pairing of Spring and Tomcat provide a simple yet powerful web application environment that avoids the use of more sophisticated, and harder to administer, application servers.

Screensaver's user interface is based upon the JavaServer Faces and Facelets web development technologies, allowing its web pages to be defined in terms of reusable templates and high-level components. For users, this results in a consistent "look and feel" across the application. For developers, the web page definitions are concise and easily interpreted, which facilitates modification. Finally, as JavaServer Faces allows its user interface components to "bind" their data contents directly to the domain model abstraction, the user interface can be readily updated in response to domain model changes.

### Data Tables

Data tables are employed throughout Screensaver's user interface. As data tables are implemented in terms of a reusable UI component, each instance provides a consistent, feature-rich user interface element for browsing, filtering, and exporting data of a particular type. Data tables provide the user with paging and sorting controls, multi-column searching/filtering (with equality, ranking, and similarity operators), column selection (optionally organized into hierarchical groups), and data export to applicable file formats (e.g., Excel workbooks, SD files, etc.).

Internally, the data table framework allows each column to be defined in terms of the domain model entity property (or properties) it represents. This allows developers to quickly update data table declarations to reflect changes in the model, and also allows the framework to automatically generate the database queries needed to populate a table's contents. The model-based column bindings are strongly typed, allowing the data table user interface to provide type-safe filtering and sorting operations for numeric, textual, and Boolean data, controlled vocabularies, and other special-case fields, such as well volumes and reagent concentrations. Finally, the framework scales to handle large data sets by loading only viewable data on-demand and minimizes the computational expense of filtering and sorting over large data sets by delegating these functions to the database layer, when necessary.

A data table can present data from multiple, related entity types, providing the user with sophisticated, customizable "views" into the domain model. For example, for an RNAi library, Screensaver's "Library Contents Browser" data table joins together entity properties from the library's wells, the RNAi reagent in each well, the gene targeted by each RNAi reagent, and experimental result data from any screen that tested the well's reagent. Note that by supporting side-by-side viewing of screen result data from multiple screens, this data table functions as a general mechanism for comparative analysis.

### User Permissions

As Screensaver is intended to service a broad group of users, including screeners and staff, it has been designed to provide selective access to data and associated functionality using a role-based system. User interface functions are exposed or hidden, as necessary, based upon a user's assigned data access roles. Staff members may be granted roles that control the types of data they are allowed to create and modify. Similarly, screeners may be granted data access roles that control the extent of data that they may access from others' screens.

To calculate data accessibility rights from user roles, Screensaver provides a pluggable data access policy mechanism where data access rights are enforced on a per-entity instance basis. As these policies are defined via Java code, sophisticated policies can be implemented by developers that take into account both user roles and the state of data. For example, the data access policy currently in use at ICCB-Longwood/NSRB supports varying levels of mutual data sharing among screeners, such that screens of a given data sharing level are made accessible to users with sufficient data access roles. This creates a tiered data access policy where screeners that are willing to share more aspects of their own screening data (e.g., raw data, hit lists, or assay protocols) are allowed to access similar levels of data from other screens.

Screeners that prefer to keep their data private are also accommodated and, similarly, do not have access to others' data and protocols.

## Results and Discussion

Screensaver features can be partitioned into the following categories that, by design, mirror the primary entity types of the underlying domain model: Users, Libraries, Screens and Studies, Cherry Pick Requests, and Activities (Figure 1). Each of these primary entity types may be browsed or searched via the standard data table user interface, while the creation, viewing, and editing of entity details is accomplished via separate "viewer" pages.

### Users

Screensaver manages two categories of users, screeners and staff. All user accounts are associated with data access roles. Roles for screeners control the types and levels of data access that are available to them. Roles for administrators are used to selectively provide data modification permissions to specific staff members, helping to ensure data integrity and enforce the facility's workflow conventions. A special "Screensaver User Login" role is used to grant or revoke login privileges to both screeners and administrators.

**Figure 2:**
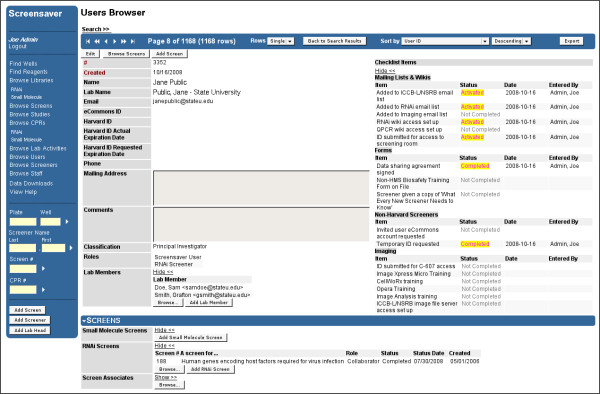
**Users Browser**. The Users Browser, available via the "Browse Users" and "Browse Screeners" menu options, allows facility staff to review the status of screeners, along with their screening projects, and their relationships to other screeners. All relevant contact information is displayed (upper left) and may be edited from this view after invoking the "Edit" command. The list of "Roles" provides a mechanism for controlling the data access permissions for the user. The "Lab Name" field, along with the "Lab Members" list, shows the members of the user's lab. The "Checklist Items" (upper right) allow staff to track the completion or activation status of administrative tasks. The (collapsible) "Screens" panel (bottom) shows the user's screening projects, partitioned into separate lists for Small Molecule and RNAi screens. The "Screen Associates" displays the full list of collaborators, lead screeners, and lab heads that are associated with the user's screening projects. A new screening project can be added from this view by invoking one of the "Add Screen" buttons. As for all data types in Screensaver, the navigation bar (top) and "Search" link allows staff to browse through users, within the context of a particular search result, ordered on a particular field. The main menu panel, shown here, is elided in subsequent figures. Note that this Users Browser page contains many Harvard-specific fields, including user ID numbers and checklist items. As for all fields in Screensaver, field names and value types can be customized by developers to the specific needs of any screening facility.

For screeners (Figure 2), Screensaver maintains contact information, relevant ID numbers, user classification (Principal Investigator, Postdoc, Graduate Student, Technician, etc.), lab affiliation, and a list of project collaborators. A system-wide configurable list of "checklist items" is also maintained for each screener, allowing the facility to track the status of any number of required administrative tasks such as account setups, required equipment and safety training, and inclusion on mailing lists. Finally, Screensaver tracks the user's associated screens and the corresponding lab activities (see below).

### Libraries

**Figure 3:**
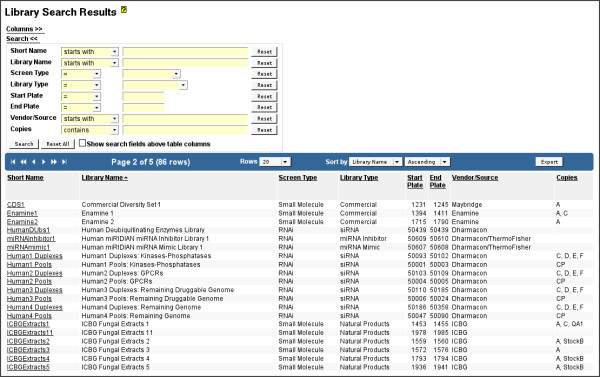
**Library Search Results**. Library Search Results, available via the "Browse Libraries" menu options, showing a summary list of the complete set of libraries maintained at the facility. In this example, the "Search" panel is shown expanded, allowing users to locate particular libraries based upon pertinent criteria. Administrator users (facility staff) will have access to additional "administrative" data columns, such as "Copies", which shows the names of library copies available for screening. Clicking on a library name will display more detail for the selected library.

**Figure 4:**
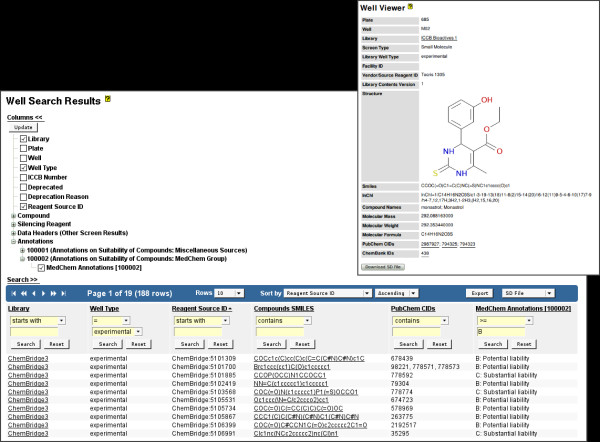
**Well Search Results**. Well Search Results, showing experimental wells for a library. The user interface for column selection is shown above, with the search user interface shown within the data table header. Note that in this case "MedChem Annotation" data from study 100002 has been appended as a data column. Similarly, by selecting available data columns under the "Data Headers (Other Screen Results)" column selection subtree, a user may compose a custom data table view that allows multiple screen results to be compared side-by-side. The resultant data may be filtered and sorted (for example, to highlight positives), and exported for external analysis. Additional description information for each well and its contents (compound and silencing reagent information) may be optionally shown or hidden, including various reagent identifiers. Note the available "Deprecated" and "Deprecated Reason" column options, which provide a mechanism for determining whether a particular well is no longer considered appropriate for screening (due to library creation errors, contamination, etc.). From the Well Search Results page, a user can navigate to the Well Viewer page for detailed information about the library well and the reagent it contains. In this example, a well from a small molecule library is shown, which includes facility-assigned identifiers, a graphical rendering of the small molecule structure, SMILES and InChi structural representations, molecular mass and weight, and identifiers assigned by third-party databases that contain compound data (PubChem and ChemBank).

A primary function of Screensaver is to manage the set of screening libraries that are maintained at the facility (Figure 3 and 4). A "library" entity represents a set of small molecule or RNAi reagents and their layout in 96-, 384- or 1536-well microplates. The library entity includes the layout of control and other special purpose wells in the library stock plates. The reagents in a given library are intended to form a cohesive set that is arbitrarily grouped by supplier, target, biological function, chemical similarity, date acquired, or any combination thereof. A given reagent may exist in multiple libraries.

Screensaver provides functionality to import, query, view, and export detailed information about each small molecule and RNAi reagent. For both reagent types, Screensaver maintains vendor information and plate/well locations. For small molecule reagents, Screensaver additionally maintains common chemical names, structural representation (in SMILES [19] and InChi [20] format) and molecular mass. It also maintains externally-assigned identifiers such as PubChem Compound IDs (CIDs) [6] and ChemBank IDs [10], with appropriate links to the respective online resources. For RNAi silencing reagents, information about the targeted gene is stored, including Entrez Gene [21] and GenBank [22] identifiers, as well as targeted gene sequences.

From the Well Search Results page (Figure 4), a user may locate reagents by querying on any of the reagent entity properties. Users can also perform a batch search for a *set *of wells or reagents in a single operation, by providing a set of plate/well designations or vendor reagents identifiers, respectively. The well data can then be exported as an Excel workbook or SD file for further analysis or communication of screen results. Details about well contents can be viewed in the Well Viewer (Figure 4 inset), including graphical rendering of small molecule structures.

The technical considerations of library storage and use (e.g., minimizing freeze/thaw cycles) dictate that library reagents be aliquoted into working copies, for use in the screening facility. Screensaver therefore tracks the copies that have been produced for each library. To handle considerations of library reagent aging and obsolescence, both individual library plates and individual reagent wells can be arbitrarily retired. When library copies are used to produce assay plates in the lab, Screensaver tracks well reagent volume usage and reports on the remaining well volumes on library copy plates. If necessary, individual well volumes can be manually updated to reflect deviations between expected and actual volumes (due to pipetting imprecision, evaporation, etc.).

Library well contents are imported from Structure Data files [23] and Microsoft Excel files for small molecule and RNAi libraries, respectively. For small molecule libraries, structure images can be imported for display on Screensaver's Well Viewer page. Screensaver provides utilities to add additional annotations to reagents (mainly external identifiers) from online resources such as PubChem, Entrez Gene, and ChemBank. If updated data becomes available for a library, Screensaver allows a new version of the library contents to be imported, while maintaining the old versions of the data for auditing purposes. This is particularly useful to handle the evolving nature of gene information, but has also aided in tracking vendor-provided corrections to well and reagent information.

### Screens and Studies

As its name suggests, Screensaver maintains a comprehensive record of the screens that are performed at an HTS facility. A key requirement for Screensaver is that it will store the screen information sufficient to interpret and reproduce each screening experiment. This sort of "minimal information" about a screening experiment is described in detail in the work of the MIBBI (Minimum Information for Biological and Biomedical Investigations) Project [24,25], in particular in the MIACA (Minimal Information About a Cellular Assay) [26] and MIARE (Minimal Information About a RNAi Experiment) [27] Projects. Screensaver does not currently fully match the MIACA and MIARE recommendations, but we are working towards that goal.

**Figure 5:**
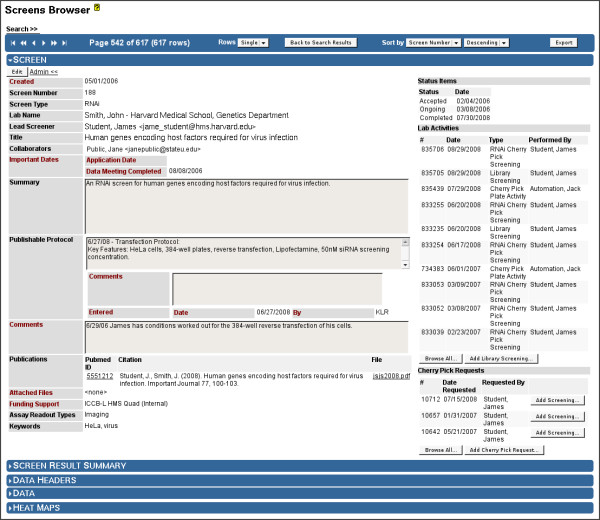
**Screens Browser: Screen Details**. The Screens Browser displays descriptive information such as screen number, screen type (RNAi or Small Molecule), title, summary, assay protocols, and keywords. Authorized staff may edit screen data, via the "Edit" command button. Associated users, including the Principal Investigator (lab name), lead screener, and collaborators are maintained for each screen. The collaborators feature also provides a means for controlling screen result data sharing among a subset of Screensaver users. Special administrative data, viewable only by staff (red labels), are maintained. These include administrative comments, attached files, and funding support information. The list of attached files allows staff to associate full documents with the screen, such as screening applications and letters of support. Publications can also be attached with citation information, which can be automatically retrieved when a PubMed ID is specified. Date-stamped screen status levels (upper right), such as "pending", "accepted", "ongoing", and "completed", provide a basic history of the progression of the screening effort. Lists of recent lab activities and cherry pick requests are shown in tables on the right, with commands for browsing and searching the full lists, and commands for adding new activities and requests (available only to screen administrators). All information concerning a screen's result data can be viewed via the expandable panels at the bottom of the page (see Figure 6). The screen panel, shown expanded, can be collapsed if the user wishes to view the result data only.

**Figure 6:**
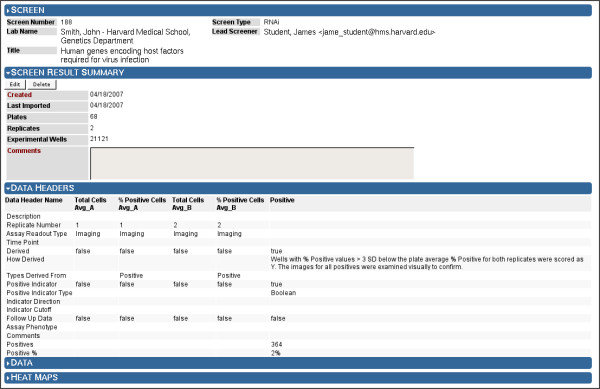
**Screens Browser: Screen Result Summary**. By expanding the "Screen Result Summary" and "Data Headers" sections of the Screens Browser, users may view the status and description of the screen result data. Since Screensaver allows result data to be imported as it becomes available, Screensaver tracks when screen result data was first created, and when it was last updated. Each screen result comprises an arbitrary set of Data Headers that describe the columns of "raw" and "derived" data, which are shown in the screen result data table (Figure 7). Derived data columns contain values calculated using values from earlier columns, and are used to record and present normalized and scored readout values, as well as the final determination of compounds identified as scoring positive in the screen (termed "Positives"). Positives data columns are treated specially, and may contain Boolean (true/false), partitioned (strong, medium, weak), or numeric values that exceed a screener-determined cutoff value. Note that for Positives data headers, users are shown the number of positives identified by the screener, both as an absolute number and as a percentage of the total number of experimental wells screened.

**Figure 7:**
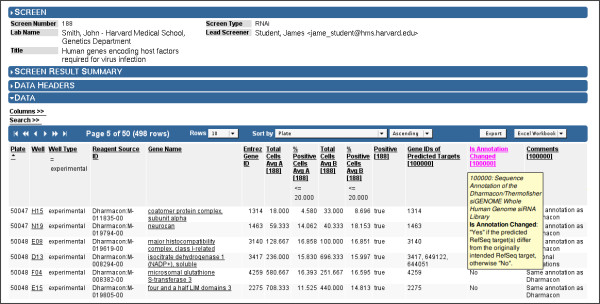
**Screens Browser: Screen Result Data**. Screens Browser, with the "Data" section expanded, showing results in a data table. Users may filter, sort, and export the data from this view. In this example from an RNAi screen, the table headers show that the search result has been filtered to display only experimental wells, with user-specified cutoff values to identify Positives scoring in both screening replicates. To gain further insights into the data, users may expand the "Columns" section to select data headers from other screen results and studies. In this example, three columns of annotation data from study 100000 have been appended. To view a detailed description of the data in any column, the user may either hover the mouse pointer over the column header to view pop-up information, or may expand the "Data Headers" section to see a full description (see Figure 6).

In Screensaver, a "screen" entity (Figure 5, 6, 7) tracks the progress of performing a screening assay, including a description of its biological significance, its experimental protocol, the raw data (from screening instrument output), and the screener-curated results. An important curated result of the screen is the set of "screening positive" reagents that have been identified as having the desired biological activity in the screening assay. Each screen entity also maintains considerable meta data in order to support the administrative needs of the facility (described in the Figure 5 legend).

Screening data are structured such that all experimental results for a given library reagent are presented in a single table row. Stored raw screen result data are quantitative. Currently, image data are not directly stored or managed by Screensaver. For each reagent, the raw data is associated with one or more data collection dimensions, presented in independent columns, that include replicate number, readout time point, and assay readout technology (Figure 6). The raw data for each library reagent are generally normalized and scored (currently via external software, e.g. common statistical and spreadsheet applications) and these intermediate analytical calculations can be added to the screen result as additional data columns. The reagents that are identified as "screening positives" are so annotated in data columns using either Boolean (true, false), "partitioned" (strong, medium, weak), or threshold-based numerical values.

**Figure 8:**
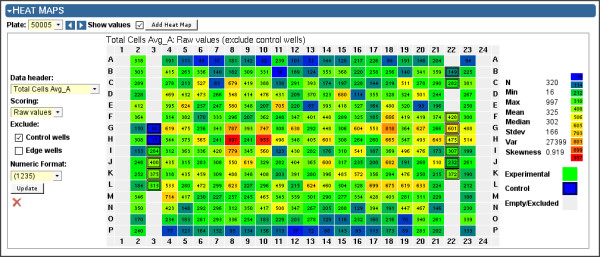
**Screen Viewer: Heat Map**. Screen Viewer, with Heat Map section expanded. A heat map can be displayed for any plate that has been screened. Multiple heat maps may be displayed side-by-side in order to compare data between alternate normalization and scoring methods. Each well (table cell) links to the Well Viewer for detailed information on the reagent being tested.

All of the above screen result data are imported into the system via the web application or a command-line utility, both of which accept a Microsoft Excel file conforming to Screensaver's screen result import file format. Once imported, all screen result data columns are viewable in a data table, which provides the expected sorting, filtering, and data export features (Figure 7). Screensaver also provides a basic facility for viewing per-plate data as a heat map (Figure 8).

In addition to screens, Screensaver provides a "study" entity type, the purpose of which is to maintain externally-available annotations for reagents, such as data provided by journal publications or public databases. The reagent annotations provided by a study allow biologically- or experimentally- relevant information to be utilized by screeners during analysis of their own screen result data. For example, a study might provide cell toxicity or fluorescence data on small molecule reagents, allowing a researcher to rule out reagents that might otherwise be considered positives. Similarly, for RNAi assays, a study might provide information about known or putative off-target effects of RNAi reagents. Study annotation data can be imported into Screensaver using the provided command-line utility, which accepts an Excel file conforming to Screensaver's study import file format.

Screensaver enables a user to make use of study data by adding one or more study annotations into a screen result data table as additional columns (Figure 7). By utilizing the data table's filtering and sorting functions, the user can assess the significance of these annotations. Similarly, data from screens that have tested common reagents can be merged into a single data table. This provides a basic mechanism for performing cross-screen comparisons, thereby leveraging the screening data being warehoused by the facility. For example, this feature may be used to compare "screening positive" reagents or reagent-specific comments between related screens. Information derived from this type of comparison might aid in the determination of whether different screens address functionally-related cellular pathways.

### Cherry Pick Requests

After a primary screen has been performed, screeners will generally perform follow-up validation assays to confirm the biological effects of screener-selected "positive" reagents. The reagents chosen for confirmation in the initial follow-up assay are "cherry picked" from library plates to form a custom sub-library plated for re-screening. The reagents chosen for this purpose constitute a "cherry pick request" and are plated in "cherry pick plates." For a small molecule screen, the number of compounds requested for cherry picking is typically 0.3%-0.5% of the total number of compounds screened in the primary screen. For genome-scale siRNA screens, cherry pick requests can include reagents for 1%-3% of the total number of genes screened.

**Figure 9:**
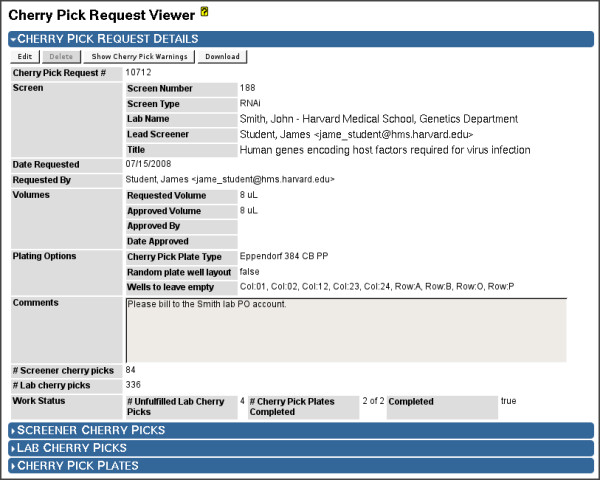
**Cherry Pick Request Viewer: Details**. Administrators use the Cherry Pick Request Viewer page to manage the creation of cherry pick plates for validation screens. The "Details" section shows the associated screen, the screener that requested the cherry picks, the date of the request, and the requested volume of reagent to be transferred for each well. An approved volume is maintained, in case the requested volume cannot be provided by the facility. The plating options allow the screener to request a randomized layout of the reagents on the cherry pick plates, and to request an arbitrary set of plate wells to be left empty (for controls, or to avoid edge effects, etc.). The remaining fields (comments, cherry pick counts, and work status fields) allow facility staff to track the status of the plate creation effort. The "Screener Cherry Picks" section (shown collapsed) contains a table of all the screener-requested cherry pick wells.

**Figure 10:**
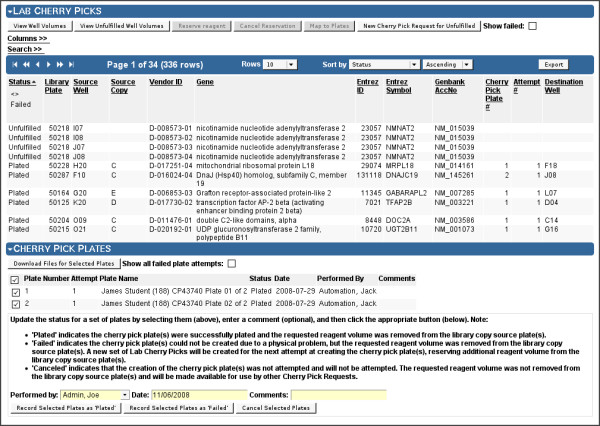
**Cherry Pick Request Viewer: Cherry Picks and Plates**. Cherry Pick Request Viewer, showing the lower "Lab Cherry Picks" and "Cherry Pick Plates" sections expanded. These sections allow the facility to manage the workflow of generating the requested assay plates. "View Well Volumes" is used to check whether sufficient well volumes exist in any of the library copy source wells, showing a table of available well volumes for each cherry pick. If so, "Reserve Reagent" is used to automatically select a library copy source well for each cherry pick well, ensuring the well of the selected copy contains sufficient volume, and decrementing the remaining volume of that well; the "Source Copy" column displays the selected copy. "Map to Plates" is then used to generate the layout of the cherry picks across one or more plates, respecting the specified layout options (see Figure 9); "Cherry Pick Plate #" and "Destination Well" columns together show the assigned plate and well on the cherry pick plate. The set of generated plates is displayed in the "Cherry Pick Plates" section. Plate mapping files, which map each cherry pick from a source copy well to a cherry pick plate well, can be downloaded for use by the lab staff. As plates are produced in the lab, administrators can update the status of each plate to "Plated", "Failed", or "Canceled". Screensaver only tracks these steps, but does not enforce or otherwise check the plating outcome.

Using the cherry pick request entity type (Figures 9 and 10), Screensaver manages the following workflow to format cherry pick plates for follow-up experiments:

1. The screener initiates the process by submitting his cherry pick list as a set of library plate/well designations, which are then are entered into Screensaver as a new cherry pick request. Screensaver reviews the set of cherry picks and enforces policies on the cherry pick count, uniqueness, and availability (e.g., non-deprecated wells). For pool-based siRNAi libraries, Screensaver automatically deconvolutes cherry picks into constituent individual RNAi duplexes.

2. Screensaver then reserves the specified volume of reagent from selected wells of library plates, ensuring that sufficient volume exists in each of the source wells. "Unfulfillable" cherry pick wells can be rolled over into a new cherry pick request for later processing.

3. A machine-readable plate mapping file is then generated to layout the reagents into the wells of one or more cherry pick plates. This file is used by automation specialists to program the liquid handling instruments to carry out the cherry picks. The plate layout can be randomized, can keep arbitrary wells empty (e.g., for controls, or to avoid edge effects, etc.), and will order and batch together reagents by source plate in order to work effectively with lab automation.

4. As the cherry pick plates are produced in the lab, staff members can update Screensaver to indicate the status of the production effort on a per-plate basis, so as to confirm (or cancel) the reservation of reagent volumes, or to indicate a physical plating failure, which will repeat the reagent reservation and plate mapping workflow for the affected plates (Figure 10).

A cherry pick request data table allows staff to efficiently determine and organize pending requests and remaining work.

### Activities

Screensaver introduces a hierarchy of "activity" entity types for tracking and auditing various events that occur in the lab and within Screensaver itself, providing a general mechanism for maintaining the "who", "what", "when", and "why" for these events. Split into two major subtypes, "lab activities" are used to represent actual events that have been performed in the laboratory by either screeners or staff, while "administrative activities" represent data-related updates and decisions made by the staff. While the hierarchy of activities is extensible, currently used types include:

• Library Screening (lab): records the act of screening a set of plates, which can be used to calculate library copy usage statistics (such as freeze/thaw counts)

• Cherry Pick Liquid Transfer (lab): records the act of creating cherry pick plates, for cherry pick request workflow management

• Well Deprecation (administrative): records the decision to flag sets of library wells as no longer being valid for screening

• Well Volume Correction (administrative): records the decision to manually adjust the volumes for a set of wells

• Checklist Item Event (administrative): records the completion or activation/deactivation of screener-associated "checklist items"

As each of the above activities are associated with a specific entity type (well, user, screen, etc.), their information appears in appropriate areas of the user interface. However, activity data are also viewable *en masse *via a data table, for general viewing and reporting purposes.

### Current Usage

Screensaver has been in production use at the ICCB-Longwood/NSRB HTS screening facility at Harvard Medical School since June, 2007. More recently, it has been implemented in screening facilities at the Netherlands Cancer Institute (NKI) [28], jointly by the A*STAR Institute of Molecular and Cell Biology (IMCB) [29] and Bioinformatics Institute (BII) [30], the High Throughput Screening & Translational Research Facility (HTS-TRF) at the Fox Chase Cancer Center [31], and the Cellular Screening Center (CSC) at the Institute for Genomics & Systems Biology (IGSB, University of Chicago) [32].

At ICCB-Longwood/NSRB, Screensaver is managing a set of 400 small molecule and RNAi screens, growing at a rate of approximately 1 to 2 new screens per week. The database contains over 45 million "raw" data values from screening instruments. More than 1,000 screeners are managed by the system, most having user accounts with login privileges. Managed libraries contain over 100,000 distinct silencing reagents (RNAi pools and duplexes are considered distinct reagents) and more than 250,000 small molecule reagents.

### Future Work

Screensaver is being actively developed and continuously improved. Because many screeners require guidance with data quality assessment and result analysis, Screensaver development efforts are shifting to address these needs. Currently, data analysis tasks are performed by external software applications, and the resultant data are simply imported into the system. However, integration of Screensaver with third-party analysis tools, such as the open source cellHTS [33] data analysis package is underway. For example, cellHTS integration will provide standardized mechanisms for scoring and normalizing raw data, as well as providing quantitative and graphical quality metrics, including normalized plate intensity, heat maps, and assay robustness metrics. Another future focus will be improved support for cross-screen comparisons, including per-reagent aggregate counts of "positives" across all screens, incorporation of validation screen results, and, potentially, dynamic incorporation of data from public databases, such as PubChem BioAssay [34], ChEMBL [9], and GenomeRNAi [8]. To facilitate data sharing with the greater HTS community, we intend Screensaver to provide functions for automating the publishing of selected data to public databases.

In response to feedback from a growing community of open source collaborators, Screensaver may become further modularized in design and deployment configurations, and may implement additional design strategies to minimize the effort needed to configure facility-specific policies and extend the data model to meet facility-specific requirements. Finally, due to the maturation and growing awareness of the MIBBI standards [24] for experimental reporting, we intend Screensaver to provide interoperability with the prescribed data formats. To this end, the structure of the screening protocols, now stored as a text (e.g., cell line, buffer recipes, transfection methods, incubation conditions, etc.), will need to be broken out into discrete fields.

## Conclusions

To support a high volume of interleaved screening projects, an HTS facility requires a sophisticated informatics and administrative infrastructure that may be best managed by a single, integrated, web-accessible application such as Screensaver. As Screensaver has proven useful in servicing the needs of the ICCB-Longwood/NSRB facility, it may provide similar benefits to other HTS facilities.

Screensaver was originally developed for the specific needs of the ICCB-Longwood/NSRB facility, however, the design of the system has already allowed other groups with appropriately skilled informatics developers to adopt and customize Screensaver to meet the needs of their HTS facility. In particular, the separation of the domain model layer from the technical infrastructure, the use of a component-based user interface framework, and the integration of a sophisticated data table component combine to make the software easy to adapt and extend. With the project team's commitment to this maintainable and extensible design, paired with the efforts of a community of facilities providing feedback and contributions via the open source development model, Screensaver will continue to accommodate the growing needs of high throughput screening facilities.

## Availability and requirements

• Project name: Screensaver

• Project home page: http://sourceforge.net/projects/screensaver/

• Operating system(s): Platform independent

• Programming language: Java

• Other requirements: Java SE 6.0, Tomcat 5.5, PostgreSQL 8.3

• License: GNU GPL2

• Any restrictions to use by non-academics: none

## Authors' contributions

ANT wrote this paper and has been developing the software since April, 2006. JPS and JLM initiated the project and developed the software through April, 2008 and April, 2005, respectively. SDE has been developing the software since October, 2008. DW, SLC, KR, SR, JN, LMS, DG, and CES contributed intellectual effort towards the design of the domain model and data workflows, aided in the determination of feature requirements, and contributed feedback for this paper. All authors read and approved the final manuscript.
